# Artificial and emotional intelligence: two key forces for teachers' professional development in an era of uncertainty

**DOI:** 10.3389/fpsyg.2026.1935683

**Published:** 2026-09-09

**Authors:** Myriam Liz Aponte, Ana Dolores Vargas Sánchez, Andrés Chiappe, Sandra Martínez-Pérez

**Affiliations:** 1 Education Faculty, Universidad de La Sabana, Chía, Colombia; 2 Education Faculty, Universidad de Sevilla, Sevilla, Spain

**Keywords:** affective data governance, artificial intelligence, relational densification, socio-emotional training, teacher wellbeing

## Abstract

The expansion of artificial intelligence in education is moving faster than institutions' capacity to protect teacher wellbeing. Current debates often oscillate between technocentric optimism, which equates success with personalisation and scale, and pre-emptive rejection driven by fears of dehumanization. This conceptual and critical synthesis proposes a different evaluative criterion: AI may support teachers' socio-emotional professional development only when it functions as relational infrastructure rather than as a symbolic substitute for human accompaniment. The manuscript introduces relational densification as a criterion for examining whether AI-supported initiatives strengthen the professional relationships associated with teacher wellbeing, including trust, mentoring, peer support, collaboration, psychological safety, and reduced isolation. It also clarifies that these relationships may have downstream implications for students through classroom climate, pedagogical responsiveness, and socio-emotional support. The article distinguishes among different AI-mediated functions, including coordination systems, recommendation tools, analytics, conversational agents, and emotional AI, and examines their differentiated ethical and pedagogical risks. Particular attention is given to affective data governance, the political economy of educational AI, cultural and linguistic specificity, and critical AI literacy as a socio-emotional competence. The article concludes that AI-supported teacher development should be assessed not only by efficiency or personalisation, but by its capacity to expand, protect, and sustain human professional relationships.

## 1 Introduction

Teacher wellbeing and the development of Emotional Intelligence (EI) can no longer be treated as a soft topic; they have become a structural matter for educational quality (Assaf and Antoun, 2024; Hidayati et al., 2023). The issue is not simply whether a teacher feels good, but whether they can sustain, over time, the affective and ethical complexity of the classroom: self-regulating under pressure, reading diversity without falling into reactivity, responding with empathy when conflict emerges, and building psychologically safe climates in which learning does not occur through humiliation or threat. Along these lines, the literature has consistently shown that teachers' socio-emotional competence is associated with better classroom climates, more effective stress management, and stronger conditions for nurturing pedagogical interactions (Arace et al., 2021; Fang et al., 2025).

Even so, a persistent gap remains between what the evidence suggests and what institutions actually implement. As mentioned by Wood (2020), training in EI is still often offered as a brief and fragmented intervention, frequently subordinated to students' social-emotional learning. Thus, the importance of the emotionally competent teacher is acknowledged, but that recognition is not matched by proportional investment in the systematic and sustained cultivation of teachers' own wellbeing. Put differently, the system declares that teachers' emotional care matters, while organizing its priorities as though it were ancillary (Hopman and Clark, 2023). This contradiction becomes even more acute when working conditions generate overload, when school management is driven by efficiency logics, and when performance evaluation privileges achievement indicators over ecologies of professional care (Fuensanta Martínez Saura et al., 2024).

Moreover, Patulny et al. (2020) indicate that in this context of high emotional demand and limited formative investment, the emergence of artificial intelligence (AI) appears simultaneously as promise and threat. On the one hand, the dominant narrative suggests that AI could enable personalized trajectories of socio-emotional development through systems capable of detecting signals of interest, frustration, or motivation, recommending activities, and even offering conversational support adapted to reported or inferred affective states (Galíndez, 2024; Moncerrate García et al., 2025; Wetcho and Na-Songkhla, 2022). On the other hand, that same promise opens a deeper dilemma: when the emotional becomes technologised, what is at stake is not only the effectiveness of a tool, but the very ontology of teacher emotional development, understood as an embodied, situated, and relational experience that is not easily reducible to algorithmic patterns (Mayer et al., 2016; Paredes Proaño et al., 2024).

This point is decisive because the development of socio-emotional competencies is not a form of technical training that can be assessed only through better responses or more accurate recommendations. In this sense, Mortillaro and Schlegel (2023) mention that developing EI does not simply mean identifying emotions and applying regulation strategies; it involves reconstructing meaning in relation with others: asking for help without shame, sustaining difficult conversations, recognizing one's limits, receiving feedback without collapsing, repairing bonds, and deliberating ethically in contexts of uncertainty. Such processes require exposure, presence, shared vulnerability, and a phenomenologically situated professional community (Trueba, 2023). Consequently, any solution that seeks to automate the socio-emotional runs the risk of degrading precisely what it intends to enhance by replacing relationship with its representation. In this regard, Alcolea López and Flórez Lozano (2025) indicate that some forms of emotional AI can simulate or classify emotional signals, but it does not have affective experience, and that ontological gap can foster illusions of reciprocity that, rather than caring, isolate.

At the same time, the opposite extreme is also intellectually unsustainable. Rejecting all technological mediation out of fear of dehumanization ignores the fact that education is already deeply mediated by technologies (platforms, metrics, analytics, and regulations) and that, when properly oriented, some mediations can ease burdens, create protected time, broaden access to resources, and support reflective practices (Farrell et al., 2024; Ortegón et al., 2024). More importantly, the relevant question is no longer whether AI enters teacher education, because that process is already underway, but under what conditions it can genuinely contribute to teacher wellbeing without eroding the relational essence of professional emotional development.

This is where the contemporary problem becomes most visible when examined through the Social Emotional Learning (SEL) framework. While public and commercial agendas tend to measure the success of educational AI by its capacity to personalize experiences, optimize resources, and scale interventions, SEL reminds us that social-emotional learning is, above all, a relational and contextual process (Foster et al., 2022; Henriksen et al., 2025). The logic of personalisation may function reasonably well when the object is informational, for instance when learners practice exercises with immediate feedback, but it becomes insufficient, and even risky, when the object is relational, as in the development of self-awareness, self-regulation, empathy, social skills, and responsible decision-making.

From the SEL perspective, therefore, socio-emotional teacher training cannot be reduced to adaptive trajectories designed algorithmically; it requires formative experiences that cultivate trust, belonging, professional recognition, and safe spaces for emotional reflection (Jones et al., 2025). Accordingly, the central question should not be only, How personalized is the pathway? but rather, How humanly dense is the formative experience that pathway makes possible? Viewed in this way, Aithal and Aithal (2023) point out that emotional training usually does not fail because of a lack of technological personalisation, but because of structural deficits in institutional culture: professional isolation, the absence of authentic mentoring, a lack of protected time for socio-emotional care, school climates that penalize vulnerability, and environments in which seeking support is interpreted as weakness. SEL, understood as an integrative framework of wellbeing, emotional competencies, and community-building, underscores that teachers' socio-emotional development cannot be sustained without meaningful relationships and without an institutional ecology that legitimizes care as a condition for learning (Fraser, 2025).

For that reason, if AI is to become part of the solution, its design cannot focus on substituting the relationship, for example, by conversing “as if” it were a human carer, but on building infrastructures that generate more human relationship: mechanisms that foster peer mentoring, densify communities of practice, activate timely support when a teacher is isolated, distribute professional care, and sustain formative conversations through ethical mediation. This is not a minor nuance. It redefines the horizon of implementation. This does not mean that personalisation is intrinsically incompatible with teacher wellbeing. Personalisation can be valuable when it increases accessibility, recognizes different professional needs, supports timely referral to human accompaniment, or helps teachers participate in mentoring and communities of practice. The problem emerges when personalisation becomes individualizing, data-intensive, and substitutive: when it optimizes profiles and recommendations while reducing opportunities for human deliberation, shared reflection, and mutual support. From this perspective, relational densification does not reject personalisation; rather, it reorients it. Personalisation becomes educationally defensible when it serves the expansion of professional relationships instead of replacing them.

The urgency of this discussion becomes clearer once we recognize that AI does not arrive on neutral ground. It enters institutions already shaped by performance pressures, by temptations of control, and by data economies that turn emotional life into an input for value extraction (Hartzog and Silbey, 2025). From this perspective, incorporating AI is never neutral; it can easily adhere to logics of optimisation and surveillance: affective data converted into raw material, predictive classification, bias in socio-emotional systems, and the displacement of affectivity into a grammar of measurement (Matos Matos, 2022). In other words, even when institutional discourse invokes “wellbeing”, actual design may end up installing emotional surveillance and performative pressure: teaching with “measurable” calm, reporting “appropriate” states, and demonstrating “balance” under metrics, as if emotional life were a KPI (Key Performance Indicator).

This risk is not only pedagogical or ethical; it is also political-economic. AI systems for education are increasingly embedded in platform markets, subscription models, data infrastructures, and public-private arrangements that shape what counts as an attractive technological solution. Recent work on platform governance in education has shown that platformisation can transfer influence over everyday educational activities to private technology companies and connect institutional decision-making to the logics of code, algorithms, and data processes (Nichols and Dixon-Román, 2024). Similarly, Ortegón et al. (2024) describe how EdTech actors and brokers participate in co-creating schools' digital infrastructures, evidence practices, and pedagogical arrangements. From this perspective, the tendency to automate socio-emotional support cannot be understood only as a design error. It is also encouraged by market incentives that reward scalability, user retention, data capture, predictive analytics, and the promise of low-cost solutions to complex institutional problems. Consequently, AI-supported teacher wellbeing initiatives should be evaluated not only by their immediate functionality, but also by the political economy of their implementation: who owns the infrastructure, who benefits from the data, how long-term dependencies are created, and whether commercial incentives align with the relational and public purposes of education.

Consequently, the debate is not truly between enthusiasm and fear, but between two incompatible formative architectures. One seeks efficiency and symbolic substitution: it automates support, reduces human accompaniment, and commodifies affectivity. The other seeks care and relationality: it uses AI to free time, coordinate community, protect data, and help institutions produce more meaningful encounters, not fewer. Thus, the article's central argument takes the form of a design thesis: in socio-emotional teacher development, AI is acceptable, and potentially valuable, when it functions as relational infrastructure rather than as a substitutive “emotional agent”; accordingly, success should be evaluated through indicators of relational densification, not by personalisation alone.

Within this framework, a distinction is made between *relational density* and *relational densification*. *Relational density* refers to the structural and experiential quality of teachers' professional relationships when these relationships are characterized by trust, emotional reciprocity, co-regulation, continuity of support, shared reflection, and collective responsibility for professional care. It does not refer merely to the frequency of interaction or to the number of contacts within a professional network. A school or teacher education programme may generate many meetings, messages, or platform-mediated exchanges without producing relational density if those interactions do not allow teachers to express vulnerability, deliberate ethically, receive meaningful support, or build sustained professional trust.

*Relational densification*, in turn, refers to the process through which those relational qualities are intentionally expanded, stabilized, and made more accessible within a professional community. In this article, the term is used as a conceptual criterion for evaluating AI-supported teacher development. An AI system would contribute to relational densification only if it helps create more protected time for human interaction, strengthens mentoring and peer-support networks, improves continuity in communities of practice, reduces professional isolation, and supports institutional arrangements that make socio-emotional care visible without turning teachers' emotional lives into objects of surveillance.

This distinction is important because the article does not claim that AI automatically improves teacher wellbeing. Rather, it proposes that AI may become educationally valuable when it supports the institutional and relational conditions through which wellbeing can emerge. In this sense, relational density is understood as a desirable condition or outcome, while relational densification names the process by which such a condition is progressively strengthened.

It is therefore not simply a matter of how frequently teachers interact, but of how bonds are configured so that legitimate vulnerability, shared ethical deliberation, accompaniment in situations of high emotional demand, and the collective construction of professional meaning become possible (Bacova and Turner, 2023). Relational density thus operates as an enabling condition for the effective development of socio-emotional competencies, in the key of Emotional Intelligence and Social Emotional Learning, and constitutes a central determinant of teacher wellbeing, even in formative contexts mediated or accompanied by intelligent technologies. From this perspective, AI does not replace relational density; rather, it must be designed to expand it, coordinate it, and protect it.

Considering the above, the article adopts an explicit position in relation to the extremes of the debate and proposes a conceptual criterion for evaluating the use of AI in teacher wellbeing. Rather than asking whether AI can develop EI on its own, the manuscript argues that teachers' professional and personal development should remain the primary mechanism for supporting teacher wellbeing. AI may contribute to this aim under specific institutional, pedagogical, and ethical conditions, particularly when it is designed as infrastructure that expands human professional relationships rather than as a symbolic substitute for them. In this sense, the central thesis of the article is propositional rather than causal: in socio-emotional teacher training, AI-supported initiatives should be evaluated not only by their capacity for personalisation, efficiency, or scale, but by the extent to which they produce demonstrable relational densification.

This formulation does not assume that AI automatically generates wellbeing, nor that relational densification has already been empirically confirmed as the causal mechanism linking AI-supported professional development to improved teacher outcomes. Instead, it advances a conceptual hypothesis for future inquiry: if AI reduces administrative burden, coordinates support, protects time for collegial interaction, and operates under strong governance safeguards, then it may help create the relational conditions associated in the literature with trust, collaboration, collective efficacy, psychological safety, and reduced isolation. The article therefore distinguishes between empirical evidence that already links these relational conditions with teacher wellbeing and the theoretical proposition that AI can support them when embedded in appropriate institutional architectures.

### 1.1 Conceptual approach and use of literature

This article is developed as a conceptual and critical synthesis, rather than as a systematic review or empirical study. Its purpose is not to estimate the prevalence of specific practices, assess intervention effects, or provide an exhaustive mapping of all research on artificial intelligence, emotional intelligence, and teacher wellbeing. Instead, the manuscript draws on selected and interconnected bodies of literature to build a theoretical argument about the conditions under which AI may contribute to teachers' socio-emotional professional development without weakening the human relationships on which such development depends.

The argument begins from research on teacher wellbeing, emotional intelligence, social-emotional learning, burnout, school climate, trust, collaboration, and collective efficacy. This literature provides the psychological and organizational basis for understanding teachers' socio-emotional development as a situated and relational process. From there, the manuscript engages with studies on AI in education, AI literacy, learning analytics, social chatbots, emotional AI, sentiment analysis, and emotion-recognition technologies. These contributions make it possible to identify the opportunities and risks that emerge when professional development becomes mediated by algorithmic systems. The analysis is then deepened through critical scholarship on datafication, affective surveillance, algorithmic bias, privacy, and educational technology governance, which helps delimit the ethical and institutional conditions under which AI should operate in this domain.

The resulting argument is interpretive and propositional. The article does not claim that relational densification has already been empirically demonstrated as a causal outcome of AI-supported teacher training. Rather, it introduces relational densification as a conceptual criterion and evaluative hypothesis for future research and implementation. This distinction is central to the manuscript. Existing literature supports associations among teacher wellbeing, trust, collaboration, burnout, collective efficacy, AI literacy, and the risks of emotional AI. The contribution of this article lies in articulating these lines of evidence into a theoretical proposition: AI may support teacher wellbeing when it is governed and designed as relational infrastructure that expands, protects, and sustains human professional relationships. Accordingly, the manuscript offers a framework for empirical testing, institutional design, and ethical deliberation, rather than a confirmed causal model.

### 1.2 Scope of AI and emotional AI in this article

In this article, AI is not treated as a single or homogeneous technology. Recent reviews have shown that educational AI includes heterogeneous applications, ranging from adaptive systems and intelligent tutoring to dashboards, automated assessment, generative AI, and emotionally responsive systems (Bond et al., 2024; Lintner, 2024). The argument developed here therefore refers to a family of systems that may participate in teacher professional development in different ways and with different levels of psychological, pedagogical, and ethical risk.

A useful distinction can be made among several AI-mediated functions. Some systems operate mainly as coordination infrastructures, for example by helping organize schedules, detect workload patterns, identify available mentoring spaces, or connect teachers with communities of practice. This type of use is close to what human-centered work on learning analytics and AI in education describes as data-intensive support that should preserve agency, safety, reliability, and stakeholder participation in design (Alfredo et al., 2024). Other systems work as recommendation tools, suggesting resources, training pathways, reflective prompts, or professional-development opportunities according to declared needs or institutional priorities. Along similar lines, Kasneci et al. (2023) note that large language models can support teachers through lesson planning, personalized content creation, differentiation, assessment, and professional development, although such opportunities need to be handled alongside risks related to bias, over-reliance, and educational judgement.

A third group includes learning analytics and dashboard systems, which aggregate traces of participation, workload, or engagement in order to support institutional decisions. These systems can be useful when they inform collective support and organizational learning, but they also require caution because learning analytics and AI in education involve issues of human control, automation, data privacy, and trustworthiness (Alfredo et al., 2024). A fourth group includes conversational agents or chatbots, which may provide guidance, reflective dialogue, or immediate responses to teachers. According to Zhang et al. (2025), natural language processing has enabled chatbot-based educational applications that interact through human language; however, evidence about affective and emotional support remains mixed and highly dependent on implementation conditions.

Finally, a more sensitive group comprises emotional AI systems, including emotion-recognition technologies, sentiment analysis, affective computing, and biometric or behavioral inference systems that attempt to detect, classify, or predict emotional states. Vistorte et al. (2024) show that AI-based emotional assessment in learning environments has been explored through techniques such as machine learning, deep learning, facial expression databases, and other forms of automated emotion analysis. More specifically, Zhang et al. (2025) define emotional AI in education as systems that detect learners' emotions, provide emotional support, or produce affective learning outcomes, while also emphasizing that more rigorous experimental research is needed to isolate the specific contribution of AI-based emotional support.

This distinction matters because the risks discussed in the manuscript do not apply equally to all these technologies. A scheduling tool that protects time for peer collaboration does not raise the same ethical concerns as a system that infers stress, motivation, or emotional regulation from facial expression, voice, text, or behavioral traces. Similarly, a chatbot that directs teachers toward human mentoring has a different pedagogical status from a chatbot designed to become an always-available emotional companion. For this reason, the critique developed in this article is not directed at AI-mediated support in general. It is directed at uses of AI that individualize wellbeing, replace human professional relationships, intensify data extraction, infer affective states without adequate safeguards, or connect emotional information to managerial and evaluative decisions.

Accordingly, the term emotional AI is used here in a restricted sense to refer to systems that claim to recognize, infer, simulate, respond to, or classify emotions through computational means. These systems deserve particular caution because they intervene directly in the socio-emotional domain and may create illusions of reciprocity, overstate the accuracy of emotional inference, or transform teachers' affective life into institutional data. By contrast, AI as relational infrastructure refers to a more limited and institutionally governed role: supporting coordination, access, continuity, referral, and participation in human professional relationships.

## 2 Design conditions and evaluation criteria for AI as relational infrastructure

The introduction argued that the debate on AI and teacher wellbeing reaches an impasse when it oscillates between two equally unproductive extremes: the promise of automating the socio-emotional and the wholesale rejection of any technological mediation for fear of dehumanization. In line with that argument, this section operationalises the article's proposal through seven design conditions and evaluation criteria that fulfill a dual function. On the one hand, they strengthen the argument by showing why AI can contribute to wellbeing only if it reorganizes the ecosystem to produce more human bonding. On the other hand, they also work as critical counterarguments, because they help identify when an implementation, despite good intentions, may erode psychological safety, displace relationships, and intensify dynamics of control.

To make the above more operational, relational densification can be examined across three complementary levels of analysis. At the individual level, it concerns teachers' perceptions of being supported, recognized, accompanied, and able to seek help without fear of stigma or punitive consequences. At the dyadic or small-group level, it refers to the quality and continuity of mentoring, peer feedback, co-regulation, and professional dialogue among colleagues. At the organizational level, it involves institutional indicators such as protected time for collaboration, sustained communities of practice, distributed leadership for care, collective efficacy, network cohesion, and formal safeguards that separate socio-emotional support from performance evaluation.

These levels also suggest different methods of assessment. Individual perceptions may be explored through validated scales on perceived social support, psychological safety, teacher wellbeing, burnout, and professional agency, complemented by interviews or reflective narratives. Dyadic and group-level processes may be assessed through mentoring logs, peer-dialogue records, qualitative analysis of professional conversations, and social-network analysis focused on the strength, reciprocity, and continuity of support ties. Organizational conditions may be examined through policy analysis, workload data, schedules of protected collaboration time, participation in communities of practice, and longitudinal indicators of trust, isolation, collective efficacy, and retention. Such indicators should not be used to monitor teachers emotionally, but to evaluate whether the institutional ecosystem is becoming more capable of sustaining professional care.

This operational clarification transforms relational densification from a general aspiration into an evaluable design criterion. It allows researchers and institutions to ask whether an AI-supported initiative uses personalisation to widen access to human support, mentoring, and professional community, or whether it merely increases platform use, individual recommendations, emotional profiling, or administrative efficiency without strengthening the relational conditions associated with teacher wellbeing. This clarification also prevents a false opposition between personalisation and relational densification. In AI-supported teacher development, the two criteria may coexist when personalisation is subordinated to relational purposes. For example, an AI system may personalize access to resources, suggest relevant communities of practice, identify voluntary mentoring opportunities, or adapt reminders for protected collaboration time without becoming an emotional substitute or a surveillance mechanism. In such cases, personalisation functions as a means of enabling relational participation. By contrast, personalisation becomes problematic when it isolates teachers into individualized trajectories, infers emotional states without adequate safeguards, or treats wellbeing as a private adjustment problem detached from workload, institutional culture, and professional community.

To clarify the practical meaning of AI as relational infrastructure, the seven design conditions proposed in this section are translated below into a more applied framework. The purpose is to show how each condition responds to a specific risk, what type of institutional safeguard would be needed, and which indicators could help evaluate whether relational densification is actually taking place. This translation is important because AI-supported teacher development should not be assessed only through platform use, number of recommendations, user satisfaction, or efficiency gains. It should also be examined in terms of its capacity to protect time for professional care, connect teachers with meaningful human support, prevent emotional surveillance, and strengthen the relational conditions associated with wellbeing.

For example, an AI-supported platform could analyse non-affective workload indicators, such as number of courses, administrative deadlines, meeting schedules, or accumulated assessment tasks, to identify periods of overload and recommend protected peer-support time. Another system could help teachers voluntarily find mentors or communities of practice according to declared professional interests, career stage, or pedagogical challenges, without inferring emotional states or creating psychological profiles. A conversational agent could also be used in a limited way to orient teachers toward available human support, suggest reflective questions before a mentoring session, or help prepare a professional-development plan, provided that it does not present itself as an emotional companion. These examples illustrate the distinction between AI that substitutes socio-emotional relationships and AI that helps institutions organize the conditions for those relationships to occur.

Table 1 synthesizes this distinction by linking each design condition with the main risk it addresses, the institutional safeguards required, and possible outcome indicators. The table should be read as a heuristic for design and evaluation rather than as a fixed measurement protocol, since indicators may vary depending on the educational setting, institutional resources, and the type of AI system implemented.

**Table 1:** AI as relational infrastructure: design conditions, risks, safeguards, and possible indicators.

Design condition	Main risk addressed	Institutional safeguard	Possible outcome indicators
Relational reinvestment of freed time	Efficiency gains are converted into more workload rather than care	Formal policy requiring that time saved through AI-supported processes be reinvested in mentoring, peer dialogue, collaborative planning, or communities of practice	Protected collaboration time; frequency of mentoring sessions; perceived time for professional care; workload balance; teacher-reported recovery time
Community as the unit of design	AI personalizes support but leaves teachers isolated in individual trajectories	Design AI systems to connect teachers with peers, mentors, communities of practice, and collaborative professional-development opportunities	Network cohesion; participation in communities of practice; peer-support ties; perceived belonging; collective efficacy
Limits on symbolic substitution	Conversational agents simulate care and displace human accompaniment	Require conversational systems to route teachers toward human support rather than acting as emotional companions	Referral to mentoring; uptake of peer-support opportunities; reduced dependence on chatbot interaction; teacher perceptions of human accompaniment
Affective data governance	Emotional or behavioral data are used for surveillance, performance evaluation, or managerial control	Apply data minimization, purpose limitation, informed consent, opt-in/opt-out mechanisms, access controls, retention limits, human oversight, and contestability	Transparency of data practices; percentage of voluntary participation; number of contested inferences; separation from evaluation systems; teacher trust in data governance
Critical AI literacy as socio-emotional competence	Teachers over-delegate judgement, naturalize recommendations, or misunderstand system limits	Include AI literacy training focused on bias, data use, emotional inference, automation limits, and relational risks	AI literacy scores; teacher capacity to explain system limits; critical use of recommendations; perceived agency in AI-supported professional development
Cultural fragility of algorithmic empathy	AI misreads culturally situated emotional expressions and produces harmful or invalidating interpretations	Avoid diagnostic claims; require contextual human interpretation; test systems across cultural and institutional settings; document known limits	Reported emotional misclassification; teacher perceptions of fairness; cultural validity checks; documented system limitations; qualitative accounts of psychological safety
Demonstrable relational densification	Success is measured only by platform use, personalisation, or satisfaction	Evaluate whether AI strengthens trust, mentoring, collaboration, support, collective efficacy, and reduced isolation over time	Trust climate; perceived social support; psychological safety; mentoring continuity; reduced isolation; burnout indicators; retention; longitudinal wellbeing measures

The table does not propose a universal measurement protocol. Rather, it translates the article's conceptual thesis into an applied heuristic for design, implementation, and evaluation. Depending on the educational setting, some indicators may be assessed through validated scales, interviews, social-network analysis, institutional records, workload data, or longitudinal mixed-methods designs. What matters is that evaluation should move beyond platform-centered metrics and examine whether AI-supported initiatives actually increase the relational conditions through which teacher wellbeing can be sustained.

### 2.1 Relational reinvestment of freed time

It is often assumed that if AI reduces administrative load or streamlines repetitive tasks, teacher wellbeing will improve almost automatically (Hashem et al., 2023). Yet that leap is problematic, because efficiency is not the same as care. Automation may open a condition of possibility, but it does not guarantee the result: it frees time, but it does not determine what that time will become. What is decisive, therefore, is an explicit institutional rule of relational reinvestment. In this sense, the time recovered through AI must be converted into protected spaces for mentoring, shared pedagogical reflection, peer accompaniment, and the strengthening of communities of practice (Medina Vargas et al., 2025). When such reinvestment is absent, “efficiency” is usually reabsorbed as increased demand, and stress is not reduced but merely reconfigured.

This point resonates with evidence from teacher wellbeing interventions showing that sustainable effects depend less on episodic actions than on consistent changes in working conditions and support structures. In that regard, Beames et al. (2023) show that interventions targeting teachers' mental health and wellbeing tend to yield better results when they are linked to conditions that can be sustained over time, rather than being limited to strategies detached from the work context. AI, then, can only be considered “fundamental” for leveraging wellbeing when its gains are translated into relational practices that were previously unfeasible because of a lack of time, not when it merely produces short-term operational relief.

Once it is accepted that freed time must be redirected toward human bonding, the next question becomes almost inevitable: what kind of relationship should be intensified, and how can it be designed so that it does not depend solely on individual voluntarism? For that very reason, the next condition shifts the unit of design from the individual to the professional fabric.

### 2.2 From the individual to the network: community as the unit of design

Personalisation almost always begins with the individual, in other words, what this teacher needs and what resource should be offered in response. Yet emotional regulation and professional wellbeing in schools and universities do not unfold as exclusively individual trajectories. As mentioned by Bjugstad et al. (2025), they are sustained, rather, by climates of support, trust, collaboration, belonging, and collective efficacy. Therefore, if the proposal advanced in this article takes relational densification as the criterion of success, AI must be designed to activate networks, not merely to optimize profiles. This means that systems should be capable of facilitating peer mentoring, connecting teachers with complementary needs, maintaining continuity between face-to-face meetings, and reducing isolation through micro-structures of support that endure over time.

This is neither a romantic nor a merely decorative idea, because evidence already links trust and collaboration with teacher wellbeing. Studies such as Tsuyuguchi (2025) suggest that a climate of trust and collaboration among colleagues is associated with wellbeing, and that collective efficacy may mediate that relationship. In turn, recent meta-analyses have shown links between collective teacher efficacy and burnout, reinforcing the importance of viewing the network as the unit of analysis when discussing wellbeing (Yurt, 2022). Consequently, a network-centered design enables AI to perform a genuinely infrastructural role: not to “care for” the teacher as a substitute for what the institution should do, but to help the institution create the conditions for mutual care.

Once AI is oriented toward networks, however, a delicate tension comes into view. In trying to “accompany” the user, some systems offer a symbolic substitute for relationship that can begin to compete with real human bonding. That is why the third condition addresses one of the quietest risks in this field: the pseudo-bond.

### 2.3 Avoiding the pseudo-bond: limits on symbolic substitution

A major risk of socio-emotional AI is not always a technical error, such as an imperfect recommendation or an inaccurate classification. Often, the deepest harm emerges when the system offers something that resembles a relationship, but in practice displaces it in the form of an “empathetic” chatbot, an emotional tutor, a companion who is always available. The problem is not that speaking to an interface is always useless, but that it can become the preferred option precisely because of its low friction, allowing users to avoid the human complexity that makes socio-emotional growth possible–negotiating, repairing, listening to what is uncomfortable, or sustaining disagreement without rupture.

Research on social chatbots is especially illuminating here. Pentina et al. (2023), for example, report how people can develop meaningful relationships with social chatbots, including dynamics of intimacy and attachment, while Possati (2023) discusses “Replika” as a case in which sustained interaction acquires complex affective layers. Translated into teacher training, the risk is paradoxical: technology is introduced in the name of wellbeing, but the result may be a growing preference for simulated bonds that reduce exposure to real intersubjectivity.

Consequently, a design consistent with the argument of this article must introduce pedagogical and ethical limits to the illusion of reciprocity. The point is not to prohibit all conversational interaction, but to prevent the interface from occupying the symbolic place of human accompaniment. Work on AI-mediated emotional connection also suggests that technological mediation should be treated as a bridge rather than a replacement for human relationships (Pankhuri and Aghamohammadian, 2024). In teacher development, conversational and recommendation systems should therefore facilitate conversations among colleagues, route teachers toward mentoring when signs of isolation appear, and promote community spaces that can sustain reflection. This condition also connects directly with the next one, because the pseudo-bond is often underpinned by the capture and inference of affective states, which opens the debate on emotional data and surveillance.

### 2.4 Affective data and governance: preventing emotional surveillance

When emotion becomes data, wellbeing can mutate into affective governance. Even when intentions are good, any system that captures, infers, or stores emotional information creates a field of risk involving classification, prediction, automated intervention, and, in the worst-case scenario, institutional control. This concern applies especially to emotional AI systems based on emotion recognition, sentiment analysis, affective computing, or biometric and behavioral inference. In the domain of so-called emotional AI, it has already been warned that emotion recognition and soft biometrics can normalize the surveillance of affective life under apparently consensual privacy arrangements and can culminate in subtle forms of control (McStay, 2020). When this is considered alongside the broader framework of surveillance capitalism, the risk ceases to be hypothetical and becomes a structural tendency of data ecosystems (Crawford et al., 2024). This is particularly relevant when wellbeing platforms operate through business models that depend on data extraction, institutional lock-in, subscription-based services, or predictive analytics marketed as efficient solutions to complex relational problems.

As the manuscript already proposes, an approach of critical complementarity requires prohibiting punitive, evaluative, or covert uses of surveillance, so that emotional information does not become an instrument of institutional control (Viac and Fraser, 2020). Along the same lines, Mohammad (2022) argues that emotion recognition systems and sentiment analysis require explicit frameworks that document their limits, biases, and risks, precisely because they tend to overstate certainties that they do not in fact possess. If the criterion of success is demonstrable relational densification, then analytics should not be oriented toward “evaluating” teachers emotionally, but toward activating timely human support through data minimization and ethical traceability.

A minimum governance framework is therefore needed whenever AI-supported teacher-development systems involve affective, behavioral, or socio-emotional data. Recent work on responsible and human-centered AI in education has emphasized that privacy, security, agency, autonomy, transparency, intelligibility, fairness, and human control are not peripheral technical issues, but core conditions for educational legitimacy (Nguyen et al., 2023). Such a framework should begin with data minimization. Systems should collect only the information that is strictly necessary to support a clearly defined pedagogical or organizational purpose, and emotional inference should be avoided when coordination, self-report, or voluntary professional dialogue can fulfill the same function with lower risk. Purpose limitation is equally important. Data gathered to support teacher wellbeing should not be repurposed for productivity monitoring, disciplinary processes, ranking, employment decisions, or performance evaluation. In socio-emotional professional development, the boundary between support and control must be explicit, auditable, and institutionally protected.

Voluntary participation and informed consent are also essential. Alfredo et al. (2024) show that human-centered learning analytics and AI in education require explicit consent, opt-in and opt-out processes, user control over data access, and explanations of how systems operate. Teachers should know what data are collected, how they are processed, who can access them, for how long they are retained, and what decisions, if any, may be influenced by them. Consent should not be reduced to a formal acceptance of platform terms, especially in institutional contexts where teachers may feel pressured to participate. For this reason, any AI-supported wellbeing initiative should include meaningful opt-in and opt-out mechanisms, clear alternatives for those who do not wish to share affective or behavioral data, and explicit guarantees that non-participation will not produce professional disadvantage.

Governance should also include human oversight and contestability. As noted in recent ethical reviews of AI in educational contexts, responsible AI requires attention to accountability, transparency, human agency, data governance, bias, surveillance risk, and privacy protection (Gouseti et al., 2025). Automated classifications, alerts, or recommendations related to teachers' emotional states should never operate as definitive judgements. At most, they should function as provisional signals that require contextual interpretation by authorized human actors trained in ethics, confidentiality, and professional care. Teachers must be able to access, question, correct, or reject algorithmic inferences about their affective state, workload, participation, or wellbeing. This is particularly important because emotional AI and sentiment analysis can overstate what can be inferred from facial expressions, voice, text, interaction traces, or behavioral data. Indeed, Vistorte et al. (2024) show that AI-based emotion assessment in learning environments relies on diverse computational techniques, including machine learning, deep learning, facial-expression analysis, and other forms of automated affective inference, which reinforces the need for caution, transparency, and contextual interpretation.

Finally, institutional safeguards should include access controls, retention limits, transparency reports, and clear separation between wellbeing support systems and managerial evaluation systems. Emotional or behavioral data should be stored only for the period justified by the stated support purpose, and access should be restricted to roles directly responsible for teacher support, not supervision or employment decisions. This concern is consistent with emerging regulatory caution regarding emotion-recognition systems in education and workplace settings, where affective inference is increasingly treated as a high-risk or even prohibited practice under certain conditions (European Union, 2024). In this sense, relational densification requires not only more professional support, but also stronger institutional guarantees that teachers' vulnerability will not be converted into a source of surveillance, classification, or managerial power.

Even when such safeguards are in place, however, one factor ultimately determines the real direction of implementation: teachers' capacity to understand limits, biases, and side effects (Kamalov et al., 2023). That is why the next condition treats critical AI literacy as part of socio-emotional development itself, rather than as an additional technical layer.

### 2.5 Critical AI literacy as a socio-emotional competence

At first sight, one idea here may seem counterintuitive: in teacher training, AI literacy is not only a technical competence; it is also a socio-emotional competence (Ajeesh and Joseph, 2025; Oh and Ahn, 2024; Paredes Proaño et al., 2024). This is because it directly affects autonomy, agency, decision-making, and the ways in which teachers seek help or decide to make themselves vulnerable in training spaces. When the system is not understood, teachers are more likely to over-delegate, to naturalize recommendations as though they were neutral, and to develop relationships of dependence. By contrast, when critical literacy is present, teachers can appropriate the tool without surrendering either their professional judgement or their relational responsibility (Essenfelder, 2026).

Understanding AI literacy as a socio-emotional competence has direct implications for teacher education and professional development. It means that teachers should not only learn how AI systems work, what they can generate, or how they may be used for planning, feedback, or assessment. They also need to learn how AI-mediated environments affect their agency, confidence, vulnerability, dependency, and relationships with colleagues and students. In this sense, critical AI literacy includes the capacity to recognize when a recommendation should be questioned, when a conversational system is creating an illusion of reciprocity, when emotional inference may be culturally or contextually inappropriate, and when a situation requires human accompaniment rather than algorithmic mediation.

This also changes the pedagogical design of teacher professional development. AI literacy should not be taught only through technical workshops focused on tool use. It should be embedded in reflective, dialogic, and ethically oriented professional learning experiences. Teachers need opportunities to examine real AI-supported scenarios, discuss dilemmas related to privacy and affective data, evaluate the risks of over-delegating judgement, and collectively define acceptable and unacceptable uses of AI in their institutions. Such learning is socio-emotional because it involves trust, uncertainty, professional identity, responsible decision-making, and the protection of one's own and others' vulnerability. From this perspective, critical AI literacy also becomes a condition for relational densification. Teachers who understand the limits, biases, and governance implications of AI are better positioned to use technological systems as bridges toward professional dialogue rather than as replacements for it. They can ask what data are being captured, who benefits from the system, whether recommendations reinforce isolation or collaboration, and whether AI-supported processes preserve or weaken psychological safety. Therefore, AI literacy should be evaluated not only by teachers' ability to operate tools, but also by their capacity to deliberate critically, protect relational trust, resist inappropriate automation, and sustain human responsibility in socio-emotional professional development.

Recent evidence suggests that this field is still in the process of consolidation. On the one hand, reviews such as Lintner (2024) point to the diversity of AI literacy scales and to the need to clarify constructs and evidence so as to avoid superficial measurement. On the other hand, exploratory studies such as Deshen et al. (2026) suggest that teachers' AI literacy includes dimensions that go beyond instrumental use and involve understanding limits, critically evaluating systems, and making informed decisions. Therefore, an AI conceived as relational infrastructure should be accompanied by explicit professional learning processes through which teachers learn to recognize when technology supports bonding and when it displaces it, what data are captured, how they are processed, which institutional decisions may be influenced by them, and how teachers can collectively contest uses that threaten trust, agency, or professional care.

From here, the discussion moves onto even more delicate ground. Even teachers with AI literacy may face failures that are not immediately visible, because the emotional dimension is always culturally situated. For that reason, the next condition focuses on the fragility of so-called algorithmic empathy.

### 2.6 The cultural fragility of algorithmic empathy and why emotional error matters

In teacher education, a content error is often correctable. By contrast, an emotional error can leave a lasting mark, because it touches recognition, dignity, belonging, and psychological safety (Bluestein and Hierck, 2025; Fernández-Berrocal and Extremera Pacheco, 2002). Socio-emotional systems, however, tend to operate through universalist assumptions about expression, tone, affect, and emotional normality (Goswami, 2025). When those assumptions fail because of cultural bias, context, irony, or ambivalence, the system does not simply make a mistake: it can invalidate experiences, pathologise legitimate responses, or induce performative pressure. This is particularly risky for teachers, for whom emotion is intertwined with professional identity, pedagogical authority, and institutional expectations.

This fragility becomes more pronounced when AI-supported teacher-development systems are transferred across cultural, linguistic, and institutional contexts. Emotional expression is not interpreted in the same way across professional cultures, school systems, languages, or communities. Silence, hesitation, irony, disagreement, indirectness, humor, formality, or expressions of fatigue may carry different meanings depending on local norms of authority, collegiality, gender, care, conflict, and professional vulnerability. This is especially relevant because automated emotion assessment often relies on facial expression, voice, text, interaction traces, or behavioral signals that may be treated as indicators of emotional states, even though emotional inference is epistemologically fragile and context-dependent (Mohammad, 2022; Vistorte et al., 2024). A system trained or validated in one context may therefore misread teachers' affective signals in another. This is not simply a question of improving model accuracy. It concerns the cultural and institutional conditions under which emotional meaning is produced, negotiated, and recognized.

For this reason, AI systems that intervene in socio-emotional teacher development should not be implemented as context-neutral solutions. They require local validation, participatory design with teachers, linguistic and cultural adaptation, and institutional deliberation about what kinds of emotional data, if any, should be collected. In multilingual or culturally diverse settings, particular caution is needed because sentiment analysis and conversational systems may misinterpret code-switching, local idioms, politeness conventions, humor, or culturally specific ways of expressing distress and professional disagreement. Critical perspectives on AI in education remind us that educational technologies are not neutral artifacts, but socio-technical systems embedded in cultural, political, and institutional arrangements (Selwyn, 2024). Accordingly, the safer design option is not to make AI more confident in emotional classification, but to restrict its role to coordination, referral, and support for human interpretation. In this sense, cultural fragility reinforces the article's central argument: relational densification depends on situated professional communities capable of interpreting vulnerability with contextual knowledge, not on algorithmic systems that universalise emotional meaning.

This, in turn, reinforces the central claim through an additional pathway: if emotion is situated and relational, then its formative processing must remain within the human community, while AI should limit itself to facilitating organization, access, and the coordination of supports. On that premise, the final condition clarifies how the article's promise can be made verifiable, namely by specifying what it means to evaluate relational densification rigorously.

### 2.7 Demonstrable relational densification as an evaluable criterion of success

Because this article is conceptual, relational densification is presented here as an evaluative proposition rather than as an already validated causal mechanism. Existing research, including the meta-analytic evidence provided by Zhou et al. (2024) and the longitudinal multilevel study conducted by Tsuyuguchi (2025), supports the relevance of several relational conditions for teacher wellbeing, including trust, collaboration, perceived social support, collective efficacy, psychological safety, and reduced isolation. However, the specific claim that AI-supported professional-development systems can strengthen these conditions through relational infrastructure remains an empirical question. For that reason, the framework proposed in this article should be read as a set of theoretically grounded design criteria and testable hypotheses for future studies, rather than as evidence that AI implementation by itself improves teachers' socio-emotional outcomes.

Relational densification cannot remain an inspiring metaphor, because if it does, the criterion of success will quickly slide back toward what is easiest to measure, such as personalisation, platform use, or immediate satisfaction. If the claim proposed here entails a genuine shift, it must also be evaluable. Accordingly, an AI-enabled socio-emotional teacher training programme should show, through verifiable evidence, that the quality and continuity of mentoring, meaningful collaboration, perceived social support, and relational trust increase, while professional isolation decreases and indicators of wear are contained. The literature provides starting points for such operationalisation. Associations have been reported, for example, between climates of trust, collaboration, and teacher wellbeing (Tsuyuguchi, 2025), while recent quantitative syntheses have also documented links between collective efficacy and burnout (Mijakoski et al., 2022).

In practical terms, this means that AI-supported coordination, recommendation, and analytics systems must be designed with embedded evidence mechanisms. The purpose is not to surveil teachers, but to evaluate the ecosystem. These mechanisms should examine whether there are more protected spaces for meeting, whether professional reciprocity is growing, whether communities of practice are sustained, and whether the institution is improving its capacity for shared care. Under these conditions, the proposal becomes more conceptually and empirically testable.

## 3 Discussion

The framework proposed in this article contributes to educational psychology by shifting the evaluation of AI-supported teacher development from a tool-centered logic to a relational and institutional logic. Its main implication is that the psychological value of AI should not be inferred from technical sophistication, efficiency, scalability, or personalisation alone. Instead, AI-supported initiatives should be evaluated by their capacity to protect time for professional care, expand access to meaningful human support, strengthen trust and collaboration, reduce isolation, and sustain the relational conditions associated with teacher wellbeing.

This relational shift also has implications for students. Although the focus of this article is teacher wellbeing and professional development, the relational conditions that sustain teachers are not isolated from classroom life. When teachers have access to collegial trust, mentoring, shared reflection, and institutional support, they are more likely to sustain emotionally responsive pedagogical relationships, manage conflict without reactivity, and participate in school climates that promote psychological safety. Conversely, when AI-supported systems individualize teacher wellbeing, intensify monitoring, or reduce opportunities for professional dialogue, the consequences may extend beyond teachers themselves. They may affect the quality of teacher-student interaction, the emotional climate of classrooms, and the forms of care, recognition, and responsiveness available to students. For this reason, relational densification should be understood not only as a criterion for teacher professional development, but also as an indirect condition for protecting the pedagogical relationships through which students experience learning, belonging, and socio-emotional support.

This shift affects how teacher wellbeing is understood in AI-mediated environments. When wellbeing is framed mainly as an individual state, AI systems may tend to offer self-regulation resources, adaptive recommendations, alerts, or personalized pathways. These functions can be useful, but they may also relocate responsibility onto individual teachers while leaving workload, institutional culture, leadership practices, and professional isolation untouched. The relational perspective developed here suggests that AI can contribute to wellbeing only when it is connected to organizational conditions that make mentoring, peer support, shared reflection, and communities of practice possible. The framework also clarifies the relationship between emotional intelligence and artificial intelligence. Emotional intelligence involves situated human capacities for emotional perception, regulation, ethical judgement, and relational action. AI does not possess emotional experience, professional responsibility, or contextual accountability. Its role in socio-emotional teacher development should therefore remain limited and carefully governed. AI may support coordination, access, referral, documentation, or preparation for reflective dialogue, but it should not become the primary site where vulnerability is interpreted, trust is cultivated, or professional conflicts are repaired.

A further implication concerns personalisation. The manuscript does not reject personalized AI-supported pathways. Personalisation can be valuable when it helps teachers access relevant resources, join communities of practice, or connect with mentors. The problem appears when personalisation individualizes wellbeing, detaches it from institutional conditions, or transforms teachers' emotional lives into data for prediction and intervention.

Governance is equally central. Affective and behavioral data governance should not be treated as a technical appendix, but as a condition for trust. Teachers are unlikely to participate authentically in socio-emotional professional development if support systems are connected to evaluation, classification, stigma, or managerial control. Data minimization, purpose limitation, voluntary participation, informed consent, human oversight, contestability, access controls, retention limits, and separation from performance evaluation are therefore pedagogical conditions for care.

Finally, the framework has clear boundary conditions. AI cannot compensate for chronic overload, punitive accountability cultures, weak leadership, or the absence of protected time for collaboration. Future research should examine whether AI-supported coordination, recommendation, or analytics systems strengthen mentoring continuity, trust climate, perceived support, psychological safety, collective efficacy, and reduced isolation. Since the framework remains conceptual, its value lies in guiding future empirical testing, institutional design, and ethical deliberation.

## 4 Conclusions

This article has argued that AI-supported teacher development should not be evaluated only through efficiency, scalability, personalisation, or platform engagement. In the socio-emotional domain, these criteria are insufficient unless they are connected to the human relationships through which teachers sustain professional wellbeing, emotional regulation, ethical judgement, and collective care. The central contribution of the article is therefore to propose relational densification as a conceptual and evaluative criterion for examining whether AI strengthens, rather than substitutes, the relational conditions of teacher wellbeing. This criterion also broadens the educational relevance of the argument, because the relational conditions that sustain teachers may indirectly shape classroom climate, teacher-student interaction, and students' experience of care, recognition, and belonging.

The argument does not imply that AI is inherently beneficial or inherently harmful. Its value depends on the institutional architecture in which it is embedded. AI may support teacher wellbeing when it helps protect time for mentoring, coordinate peer support, sustain communities of practice, widen access to professional accompaniment, and make relational support more visible without turning teachers' affective lives into data for surveillance or performance evaluation. By contrast, AI becomes problematic when it individualizes wellbeing, simulates emotional reciprocity, intensifies data extraction, or shifts responsibility for structural problems onto teachers themselves.

This conclusion also reframes the relationship between emotional intelligence and artificial intelligence. Teachers' socio-emotional development remains an embodied, situated, and relational process. AI can support the conditions for that process, but it cannot replace the human judgement, trust, vulnerability, and institutional responsibility that make emotional learning professionally meaningful. For this reason, governance is not an external requirement but part of the pedagogical design itself. Data minimization, purpose limitation, voluntary participation, human oversight, contestability, and separation from performance evaluation are necessary to preserve the psychological safety on which relational densification depends. These safeguards become even more important when AI systems are embedded in platform markets, data-driven business models, culturally diverse institutions, and multilingual professional communities where emotional meaning cannot be treated as context-neutral.

The framework proposed here remains conceptual and should be empirically tested. Future research should examine whether AI-supported coordination, recommendation, analytics, or conversational systems can strengthen mentoring continuity, trust, perceived support, collective efficacy, psychological safety, and reduced isolation under real institutional conditions. Longitudinal, mixed-methods, participatory, comparative, and social-network studies would be especially useful for evaluating these pathways. Ultimately, AI will be educationally defensible in teacher wellbeing initiatives only when it helps institutions produce more meaningful human relationships, not fewer.
